# Bilateral Asymmetric Dislocations of Hip Joints: An Unusual Mechanism of Injury

**DOI:** 10.1155/2013/694359

**Published:** 2013-02-20

**Authors:** Rajesh Kumar Kanojia, Satya Ranjan Patra, Sumit Gupta

**Affiliations:** ^1^Department of Orthopaedics, Lady Hardinge Medical College, New Delhi 110001, India; ^2^Sparsh Hospitals and Critical Care, Bhubaneswar, Odisha 751015, India; ^3^Saraswathi Institute of Medical Sciences, Hapur, Ultra Pradesh 245304, India

## Abstract

Asymmetric bilateral dislocations of the hips are rare injuries. Among the small number of reports in the literature, most have attributed the cause to high-velocity motor crashes. These dislocations are often seen to be associated with fractures of the proximal femur or the acetabulum. We present a case of a 45-year-old man with bilateral asymmetric dislocation of hips which were purely ligamentous in nature, without any fracture. He sustained his injuries due to a fall while getting on a moving bus. It was an unusual mechanism of injury as compared to the other cases of asymmetric hip dislocations reported in published studies. Both hips were reduced under general anaesthesia within three hours of the trauma. Skin traction and non-weight-bearing rehabilitation were continued for six weeks. After 35 months of followup, the patient remains asymptomatic. Early diagnosis and timely reduction of such dislocations under anaesthesia are necessary for prevention of complications.

## 1. Introduction

Hip dislocations are common occurrences following high-velocity trauma. But simultaneous bilateral hip dislocations only constitute 1.25% of all hip dislocations [1]. Simultaneous one-side anterior and one-side posterior hip dislocations (asymmetric dislocation) are even less common [1]. Hip joint being an inherently stable joint requires a significant amount of force to dislocate [2, 3]. Determining the exact mechanism of trauma to cause bilateral asymmetrical dislocation is a difficult task. Different researchers in the past have described various modes of injuries, the most common being vehicular accident causing a deceleration injury [3–5].

In this paper, we give the account of a patient who sustained bilateral asymmetric traumatic dislocation of the hips while trying to get on a speeding bus. The mechanism of injury and the reduction manoeuvres of this unusual injury are discussed along with a short review of the literature. 

## 2. Case Report

**Figure 1:**
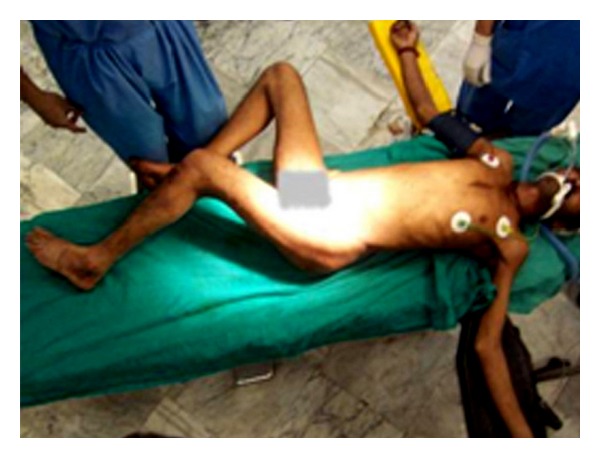
Clinical picture of the patient lying on the operation table showing the attitude of the lower limbs.

A man of 45 years of age presented to our trauma centre after sustaining injuries to both hips due to a fall while trying to get on an accelerating bus from the rear entrance. He was under the influence of alcohol when he had the accident. At the time of presentation, the attitude of his left lower limb was flexed, adducted, and internally rotated; in contrast, the right lower limb was flexed, abducted, and externally rotated (Figure 1). Multiple small abrasions and contusions were seen over both knees and left trochanteric region. On clinical examination there was no other musculoskeletal injury or neurovascular deficit in any of the limbs.

**Figure 2:**
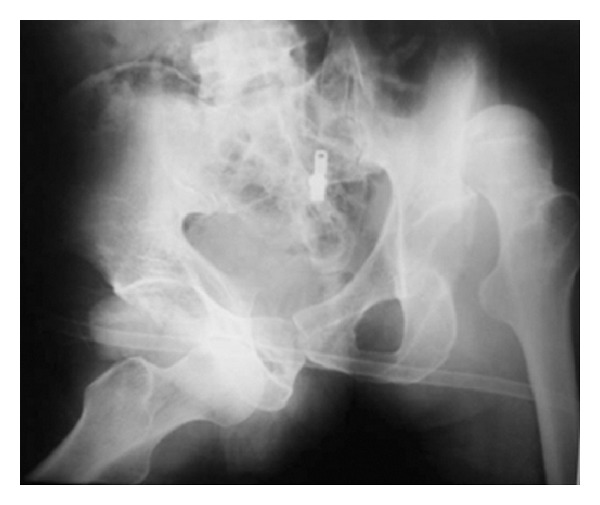
Radiograph showing anterior dislocation of right hip and posterior dislocation of left hip joint, without any associated fracture.

The radiographs showed posterior dislocation of the left hip joint (Thompson and Epstein type I) and anterior dislocation of the right hip joint (Epstein type IIA, obturator type) [6]. No fracture could be seen on radiographs in proximal ends of either femur or either acetabulum (Figure 2).

**Figure 3:**
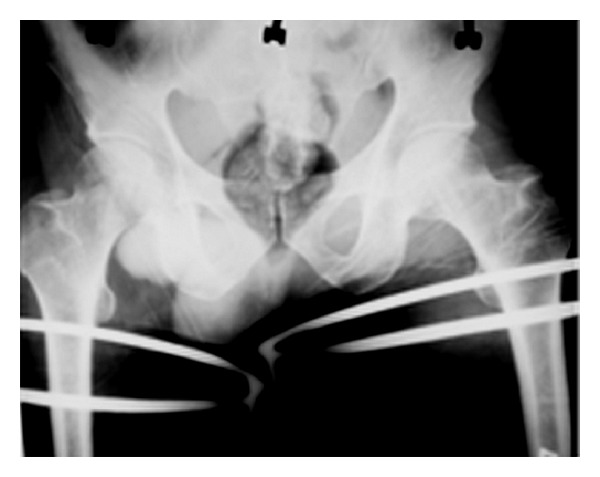
Radiograph of the pelvis of the same patient showing reduction of the dislocated hips.

Closed reduction of the joints was planned under general anaesthesia. Approximately three hours after injury, the patient was laid supine on the operating table. After the patient was properly anesthetized, closed reduction of the posterior dislocation of the left hip was done using the Allis manoeuvre [6]. The affected hip and knee were flexed to 90 degrees; traction was applied in the line of femur with the assistant holding the pelvis firmly on the table by pressing down both iliac crests. For the anterior dislocation of the right hip, a similar manoeuvre was used, the only difference being that an assistant was instructed to give lateral traction on the proximal thigh with the help of a draping towel. After reduction, the patient was immobilized on bed with skin traction applied to both lower limbs for three weeks. A repeated radiograph showed both hip joints in reduced position (Figure 3). He was advised to do static quadriceps exercises on bed. After three weeks, patient was allowed to sit up on bed, and gentle non-weight-bearing mobilization of hip and knee joints initiated. Weight bearing was started at the end of six weeks with the help of a walker. Patient was followed up regularly and was last seen 35 months following injury. He had no complaints concerning his affected joints, and he was having a normal painless gait without any limp or lurch.

## 3. Discussion

Bilateral simultaneous dislocations of hip joints are uncommon. Agarwal et al. reported it to be 1.25% of the total reported hip dislocations [1]. Bilateral asymmetric dislocations of hip are even rarer, and only limited number of cases have been reported. Because of the violent trauma involved in these injuries, these dislocations are often associated with fractures of the acetabulum, head of femur, neck of femur, trochanter, and even shaft of femur [4, 5, 7]. In contrast, our case happens to be purely ligamentous dislocation of both hips without any fracture. Only few cases of purely ligamentous bilateral asymmetric dislocations of hip joints are reported to date [2, 8].

The patient sustained injury while getting on a moving bus. We tried to analyze the most probable mechanism of his injury by correlating the sequence of events with those described in the literature by various authors. His left foot was on the pedestal and right foot was on the ground. Before he could hop off the ground and transfer his bodyweight onto his left leg, the bus accelerated. This forced his right hip to go into sudden abduction and external rotation, thereby dislocating the femoral head anteriorly. During the fall, he landed on the anterolateral aspect of his left knee with a flexed and adducted left hip, and his bodyweight and the momentum of the fall resulted in dislocation of the left hip posteriorly.

Examining the various modes of injury described in published studies for bilateral asymmetric dislocations of the hip joints, they ranged from pedestrian being hit by a car to head on collision of vehicles and motorcycle crash [1, 2, 8]. The single common mechanism involved in most of these cases was a sudden deceleration injury. In contrast, the mode of injury in the present case was found to be different and unlike that of high-velocity collisions. The lower energy of impact involved in this case was probably responsible for the purely ligamentous nature of the dislocations.

In our patient, reduction of posteriorly dislocated left hip joint was done by the commonly used Allis manoeuvre [6], but reduction of the anteriorly dislocated right hip joint was the trickier one as anterior dislocation is much less commonly encountered in clinical practice. The recommended manoeuvre is similar to the Allis method, but with an addition of lateral traction by an assistant [9]. This lateral traction dislodges the femoral head from the anteromedial soft tissues around the obturator foramen region. A similar method of manipulation was used in our case, and both hip joints could be reduced without much difficulty. In our opinion, absence of any fracture in this patient was also a cause for the easy reduction. To rule out the presence of any intra-articular loose fragment, Dudkiewicz et al. have opined in the favour of a routine postreduction computed tomography (CT) scan [4].

Both skeletal and skin traction methods have been recommended for varied durations (3 to 8 weeks) in published studies [2, 10], although there are also opinions against such prolonged immobilization of patients on bed. We preferred to keep the patient on bilateral skin traction for a period of three weeks before starting mobilization exercises.

Most researchers are of the view that incidence of avascular necrosis (AVN) of the femoral head is often determined by the delay in reduction of the dislocated hip [1–3, 5]. The risk of AVN may range from 8% to 15% in closed reduction cases and may go up to as high as 40% in cases requiring operative reduction [2]. Agarwal et al. have recommended a routine magnetic resonance imaging (MRI) scan at around three months following injury for early detection of AVN [1]. Incidence of posttraumatic arthritis has been stated to be approximately 24% in patients of hip dislocations [3–5]. 

Asymmetric bilateral hip dislocation is a rare entity. Although rapid deceleration injury is the mechanism involved in most cases, the study of this case turns our attention towards a different mode of trauma resulting in a similar injury pattern. Prompt reduction of the dislocations can prove crucial for the long-term prognosis of such injuries.
